# Cellular Cholesterol Transport Proteins in Diabetic Nephropathy

**DOI:** 10.1371/journal.pone.0105787

**Published:** 2014-09-02

**Authors:** Joseph G. S. Tsun, Susan Yung, Mel K. M. Chau, Sammy W. M. Shiu, Tak Mao Chan, Kathryn C. B. Tan

**Affiliations:** Department of Medicine, University of Hong Kong, Hong Kong, Hong Kong; National Center for Scientific Research Demokritos, Greece

## Abstract

**Background:**

Lipid accumulation has been shown to accelerate renal injury, and the intracellular accumulation of lipids may be caused by alterations in synthesis as well as lipid uptake and efflux. We have investigated the role of cellular cholesterol transport proteins including adenosine triphosphate binding cassette transporter A1 (ABCA1), G1 (ABCG1) and scavenger receptor class B type I (SR-BI) in diabetic nephropathy.

**Methods:**

Protein expression and the ability to mediate cholesterol efflux of ABCA1, ABCG1 and SR-BI was determined in human renal mesangial cells and proximal tubular epithelial cells cultured under normal or high glucose conditions. Renal expression of these cholesterol transporters was examined in a murine model of streptozotocin-induced type 1 diabetes.

**Results:**

ABCA1, ABCG1 and SR-BI were expressed in both human renal mesangial cells and proximal tubular epithelial cells, and mediated cholesterol efflux to apolipoprotein AI and HDL. *In vitro*, hyperglycemia reduced the expression and the ability to mediate cholesterol efflux of all three cholesterol transporters (*p*<0.05). *In vivo* studies showed that intra-renal accumulation of lipids was increased in diabetic mice, particularly in mice with nephropathy. This was associated with a significant reduction in the expression of ABCA1, ABCG1 and SR-BI in the kidneys. These changes were already seen in diabetic mice without nephropathy and preceded the development of nephropathy. Diabetic mice with nephropathy had the lowest level of these cholesterol transporters.

**Conclusion:**

Inducing diabetes with streptozotocin significantly reduced renal expression of ABCA1, ABCG1 and SR-BI. Defects in cholesterol export pathway in renal cells could therefore promote cholesterol accumulation and might contribute to the development of diabetic nephropathy.

## Introduction

Recent studies of progression of chronic kidney disease indicate that, like atherosclerosis, lipid accumulation can contribute to glomerular injury, and there is growing evidence that renal accumulation of lipids play a role in the pathogenesis of diabetic nephropathy [1]–[3]. Intra-renal accumulation of lipids has been observed in diabetic patients and experimental animals [4], [5], and renal lipid accumulation may accelerate glomerulosclerosis and interstitial fibrosis through lipid infiltration and induction of oxidative stress, proinflammatory cytokines, and growth factors [5]–[7]. The mechanism(s) for lipid accumulation in diabetic nephropathy is not fully understood. The intracellular accumulation of lipids and formation of lipid droplets may be caused by alterations in synthesis [5], [8] as well as lipid uptake [9], [10] and efflux.

Cellular cholesterol efflux occurs by transport mediated by specific cholesterol transport proteins in addition to aqueous diffusion [11]. Cholesterol transporters involved in cellular cholesterol efflux include adenosine triphosphate binding cassette transporter A1 (ABCA1), ABCG1, and scavenger receptor class B type I (SR-BI) [11], [12]. ABCA1 mediates cholesterol efflux to lipid-free apolipoprotein AI (apo AI) and pre-β HDL, whereas both ABCG1 and SR-BI mediate cholesterol efflux to mature HDL. There is experimental evidence suggesting that changes in cholesterol efflux may be involved in renal lipid accumulation. Ruan et al. have shown that interleukin 1 beta promotes intracellular lipid accumulation in mesangial cells by inhibiting cholesterol efflux mediated by ABCA1 [13]. Mesangial cells are specialized glomerular pericytes that share many properties with macrophages [14]. Mesangial cells metabolize lipids similar to macrophages, and they take on the appearance of foam cells after accumulation of lipids [15]. Acute renal tubular injury can also cause down regulation of ABCA1 and SR-BI [16]. Tang et al. recently reported that ABCA1 expression was reduced in the kidneys in diabetic NOD mice and was accompanied by an increase in renal cholesterol [17]. The role of ABCG1 and SR-BI in renal cellular cholesterol efflux in diabetic nephropathy has not been determined. We have therefore evaluated the contribution to cellular cholesterol efflux by the three cholesterol transporters in human mesangial and proximal tubular epithelial cells (PTC) and investigated whether renal expression of these cholesterol transporters is decreased in an animal model of diabetic nephropathy.

## Materials and Methods

### Cell culture

Primary human mesangial cells (NHMC) were purchased from Lonza (Walkersville, MD) and cultured with Mesangial Cell Growth Medium (Lonza, Walkersville, MD) supplemented with 5% FCS according to the supplier's instructions. Normal proximal tubular epithelial cells (PTC) immortalized with the human papilloma virus 16 E6/E7 genes [18], HK-2 cells, were purchased from ATCC (Manassas, VA) and cultured with DMEM/F12 medium (Life Technologies, Grand Island, NY) supplemented with 5% FCS. Cells were fed every three days until 80% confluent. Both cell culture media used contained 5 mM glucose. Cells were incubated with or without additional dosages of D-glucose to cell culture medium (final concentrations 5–55 mM) for 24 hours for further Western blot analysis or cholesterol efflux.

### Western blot analysis

At the end of the incubation with glucose, cells were lysed in M-PER Mammalian Protein Extraction buffer (Pierce, Rockford, IL), and protein concentrations were quantified by Lowry protein assay (Bio-RAD, Hercules, CA). Aliquots of total cell lysates obtained from NHMC or HK-2 cells under control or experimental conditions, or from renal cortical tissue (20 µg total protein as determined by the Lowry protein assay) were denatured with sample loading buffer containing 5% β-mercaptoethanol (Invitrogen, Grand Island, NY). Samples were then heated at 95°C for five minutes and subjected to SDS-PAGE followed by Western blot. Membranes were extensively washed with TBS-T in between all steps. Membranes were probed respectively with primary antibodies of ABCA1 (Novus Biologicals, St. Louis, MO), ABCG1 (Santa Cruz Biotechnology, Dallas, TX), SR-BI (Novus Biologicals, St. Louis, MO), CD36, SR-AI, LOX-1, LXR-α, and LXR-β (Abcam, Cambridge, UK) (diluted 1∶1,000), followed by the respective secondary antibodies conjugated to HRP (Cell Signaling Technology, Danvers, MA) (diluted 1∶2,500). Protein bands were visualized with ECL Plus according to the manufacturer's instructions (GE Healthcare, Buckinghamshire, UK).

### Cholesterol efflux

After incubating with various concentrations of D-glucose, cells at 80% confluence were labeled with tracer [^3^H]-cholesterol (1 µCi/well) for 16–20 hours at 37°C, and then equilibrated with 0.5% BSA for four hours before the addition of different cholesterol acceptors. 10 µM of chemical that blocks lipid transport (BLT-1, EMD Millipore Chemicals, San Diego, CA) was added during equilibration to inhibit SR-BI activity as indicated [19]. Apo AI (20 µg/ml, Biodesign, Memphis, TN), HDL, HDL_2_ and HDL_3_ (25 µg protein/ml, BTI, Stoughton, MA) were then used to induce cholesterol efflux from the labeled cells for six hours. Radioactivity in media and cells were quantified by liquid scintillation counting using a Packard scintillation counter (Perkin Elmer, Waltham, MA). The percentage of cholesterol efflux from cells was calculated based on the radioactivity in the media divided by the total radioactivity in cells and in media.

### Ethics statement

All animal procedures were approved by the Institutional Committee on the Use of Live Animals in Teaching and Research at the University of Hong Kong. Animal welfare was monitored daily and all efforts were made to minimize suffering. Any mouse showing signs of limited movement or significant weight loss were euthanized by intra-peritoneal injection of pentobarbitone (150 mg/kg body weight).

### Animal studies

Male DBA/2J mice at six to eight weeks of age were a kind gift from Prof Y. L. Kwong (Department of Medicine, University of Hong Kong) and received standard chow and water *ad libitum.* After one week acclimatizing to their surroundings, mice were fasted for six hours prior to intra-peritoneal injection of streptozotocin (STZ, 50 mg/kg) in 10 mM citrate buffer, pH 4.5, administered on five consecutive days. Diabetes mellitus was confirmed by tail vein blood sampling of glucose concentration, measured with Accu-Chek Advantage II Glucostix test strips. Spot urine was assessed weekly for albuminuria using QuantiChrom albumin assay kit until sacrifice. Mice with diabetic nephropathy, confirmed by proteinuria with urinary albumin excretion rate >100 mg/dl on two occasions at least two days apart were sacrificed (n = 5). Blood samples were obtained by cardiac puncture and the kidneys harvested, decapsulated and weighed. The left kidney was cut perpendicular to the long-axis and one half of the kidney was snap frozen in OCT followed by immersion in liquid nitrogen, while the second half was fixed in 10% neutral-buffered formalin followed by paraffin embedding. Renal cortical tissue from the right kidney was separated from the medulla and frozen at −80°C. Age- and sex-matched control groups included non-diabetic male DBA/2J mice, and diabetic mice without nephropathy (n = 5 per group). Serum creatinine and urea levels were measured with QuantiChrom creatinine and urea assay kits respectively, while serum triglyceride and cholesterol were measured with Biomerieux triglyceride and cholesterol kits.

### Cytochemical staining

Intra-renal expression of ABCA1, ABCG1 and SR-BI in control and diabetic mice were determined using the peroxidase-anti-peroxidase method with the same antibodies used in Western blot and counterstained with hematoxylin as previously described [20], [21]. Intra-renal staining was semi-quantitatively assessed in at least 15 separate images per mouse kidney, and the extent of staining graded by two independent workers in a blinder manner as follows: 0: 0–5% staining, 1: >5–25% staining, 2: >25–50% staining, 3: >50–75% staining, 4: >75% staining.

### Oil Red O staining

Frozen kidney sections were used for Oil Red O staining to determine the renal accumulation of neutral fats. Briefly, frozen kidney sections were first washed with tap water followed by rinses with 60% isopropanol. Sections were then stained with freshly prepared Oil Red O solution 15 minutes before nuclei staining with alum haematoxylin. Stained sections were finally mounted in glycerine jelly.

### Statistics

All experiments were carried out for three times. Numerical data were represented as mean ± standard deviation (SD). Dunnett *t*-tests were used to compare multiple diabetic groups with the non-diabetic control as the reference group. Two-tailed *p*<0.05 was considered statistically significant.

## Results

**Figure 1:**
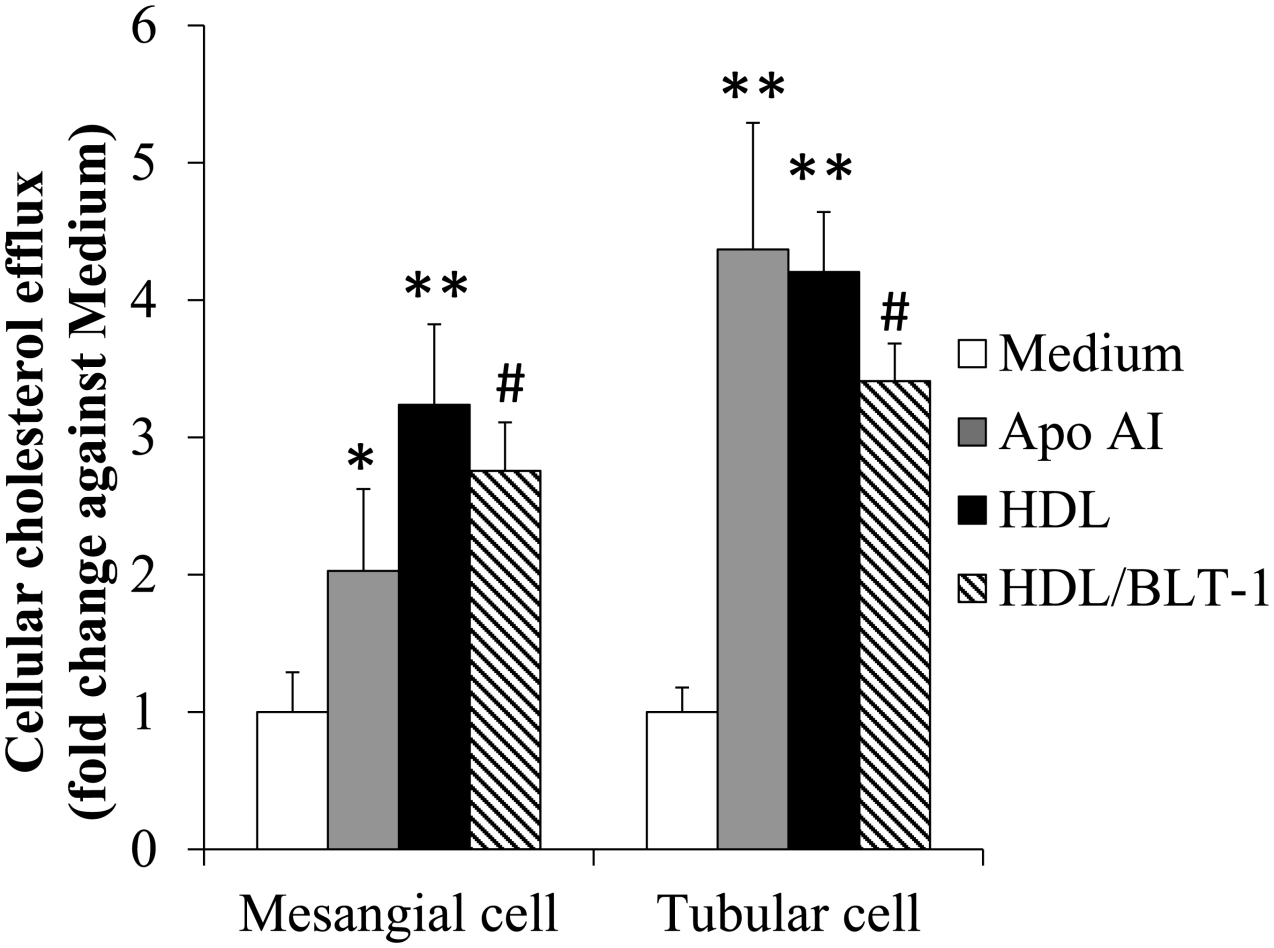
Cellular cholesterol efflux to different cholesterol acceptors under basal condition. Cellular cholesterol efflux from mesangial cells and tubular cells to apo AI, HDL and HDL with BLT-1 (HDL/BLT-1) under basal condition with 5 mM glucose in culture medium was presented as means + SD from 3 separate experiments in duplicate, *p<0.05 and **p<0.01 vs. cell culture medium without any cholesterol acceptor; ^#^ *p*<0.05 vs. HDL as cholesterol acceptor.

All three cholesterol transporters (ABCA1, ABCG1 and SR-BI) were expressed in human mesangial cells and in tubular HK-2 cells at basal state (Figure S1). Apo AI was able to stimulate cellular cholesterol efflux from both cholesterol-laden mesangial and tubular cells, suggesting that ABCA1 transporter was functional (Figure 1). Cholesterol efflux experiments were also performed with HDL, HDL_2_ or HDL_3_ as acceptor since both ABCG1 and SR-BI could mediate cholesterol efflux to mature HDL. There were no significant differences in cholesterol efflux when the same amount of HDL, HDL_2_ and HDL_3_ was used (Figure S2). The proportion of cholesterol efflux to HDL mediated by SR-BI was represented by the degree of reduction in cholesterol efflux after incubation with BLT-1 (an inhibitor of SR-BI), and the remaining cholesterol efflux would likely be mainly mediated by ABCG1 (Figure 1).

**Figure 2:**
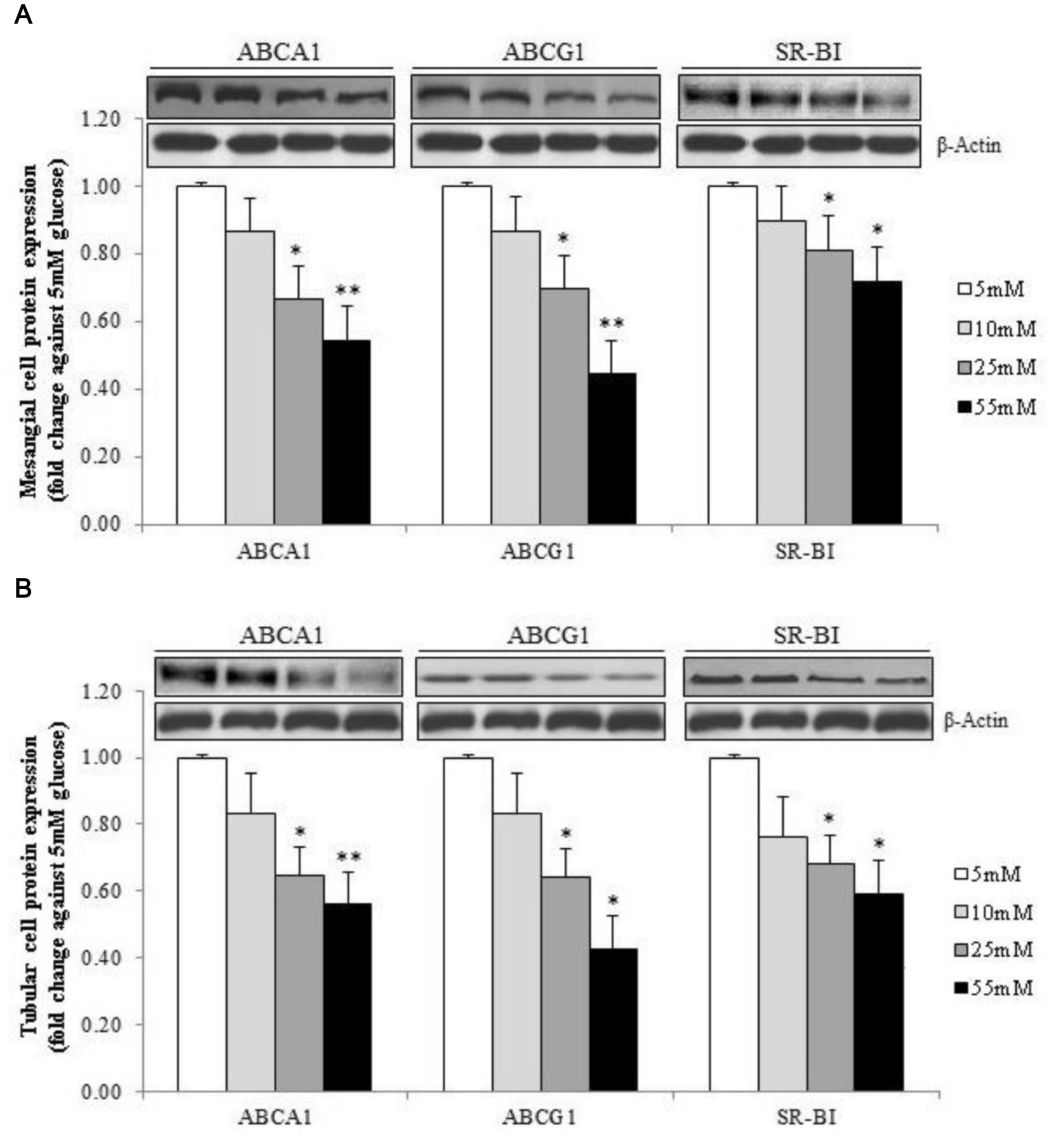
Expression of cholesterol transporters under different concentrations of glucose. Human mesangial cells (A) and HK-2 cells (B) were cultured under different conditions of glucose concentrations. Relative target protein band intensities were normalized against its *β*-actin and were presented as means + SD by densitometric analysis from 3 separate experiments. *p<0.05 and **p<0.01 vs. cells with 5 mM glucose in culture medium.

When mesangial cells and HK-2 cells were incubated under high glucose conditions, the expressions of all three cholesterol transporters were significantly decreased in a dose-dependent manner (Figure 2). This was accompanied by a reduction in cholesterol efflux to apo AI and to HDL (Figure S3), suggesting that hyperglycemia might impair cellular cholesterol efflux by reducing the expression of cholesterol transporters.

**Figure 3:**
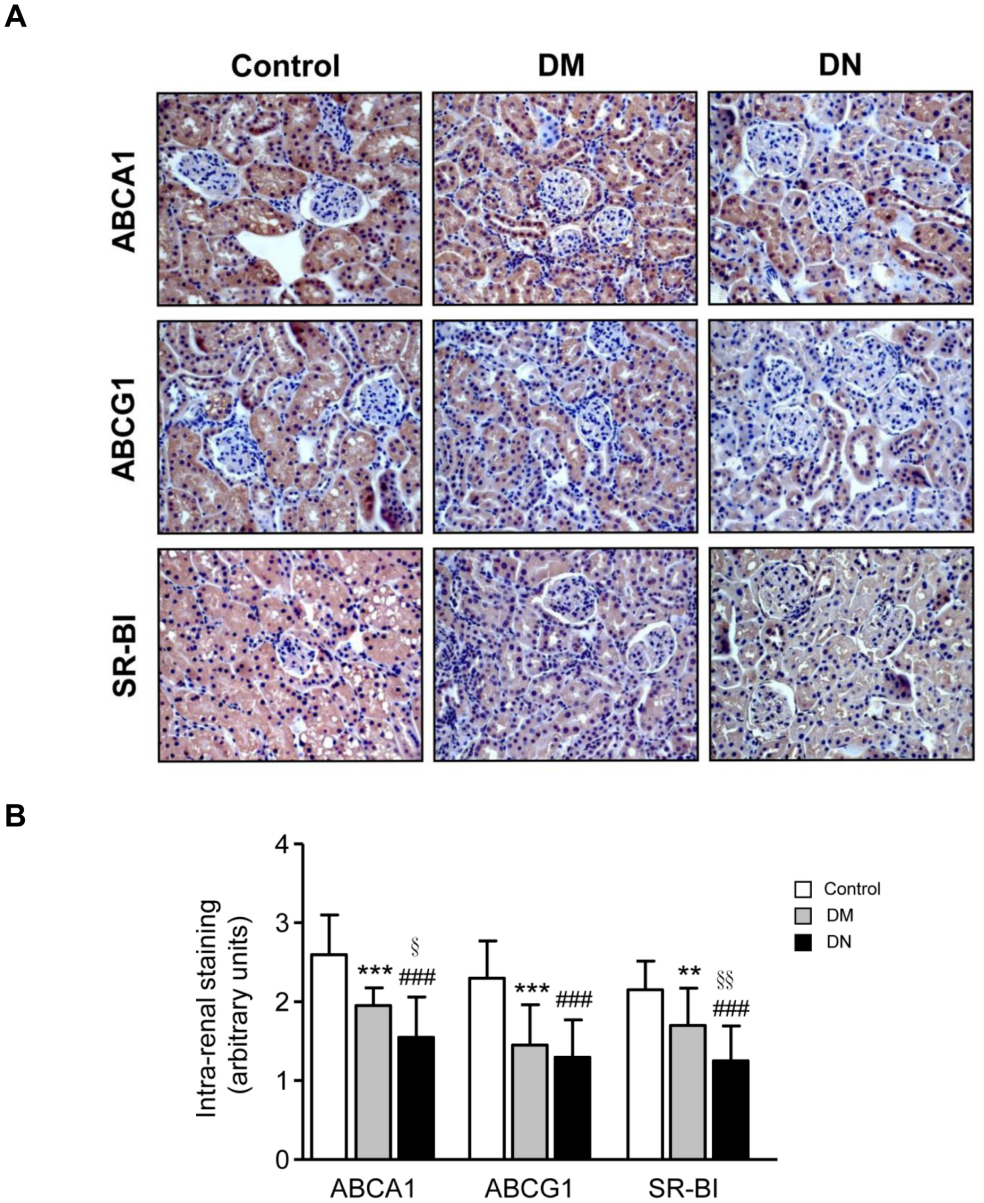
Immunohistochemical images of cholesterol transporters in different groups of mice. Representative images of cholesterol transporters ABCA1, ABCG1, and SR-BI in renal tissues from control, diabetic (DM), and diabetic nephropathy (DN) mice. Original magnification x400 (A). Image-based computer assisted analysis was performed to semi-quantify the amount of ABCA1, ABCG1 and SR-BI in the glomeruli and tubulo-interstitium of control, DM and DN mice (B). Results were presented as mean + SD of data obtained from 5 mice per group. **p<0.01 or ***p<0.001, control vs. DM; ^###^ *p*<0.001, control vs. DN; ^§^ *p*<0.05 or ^§§^ *p*<0.01, DM vs. DN.

**Figure 4:**
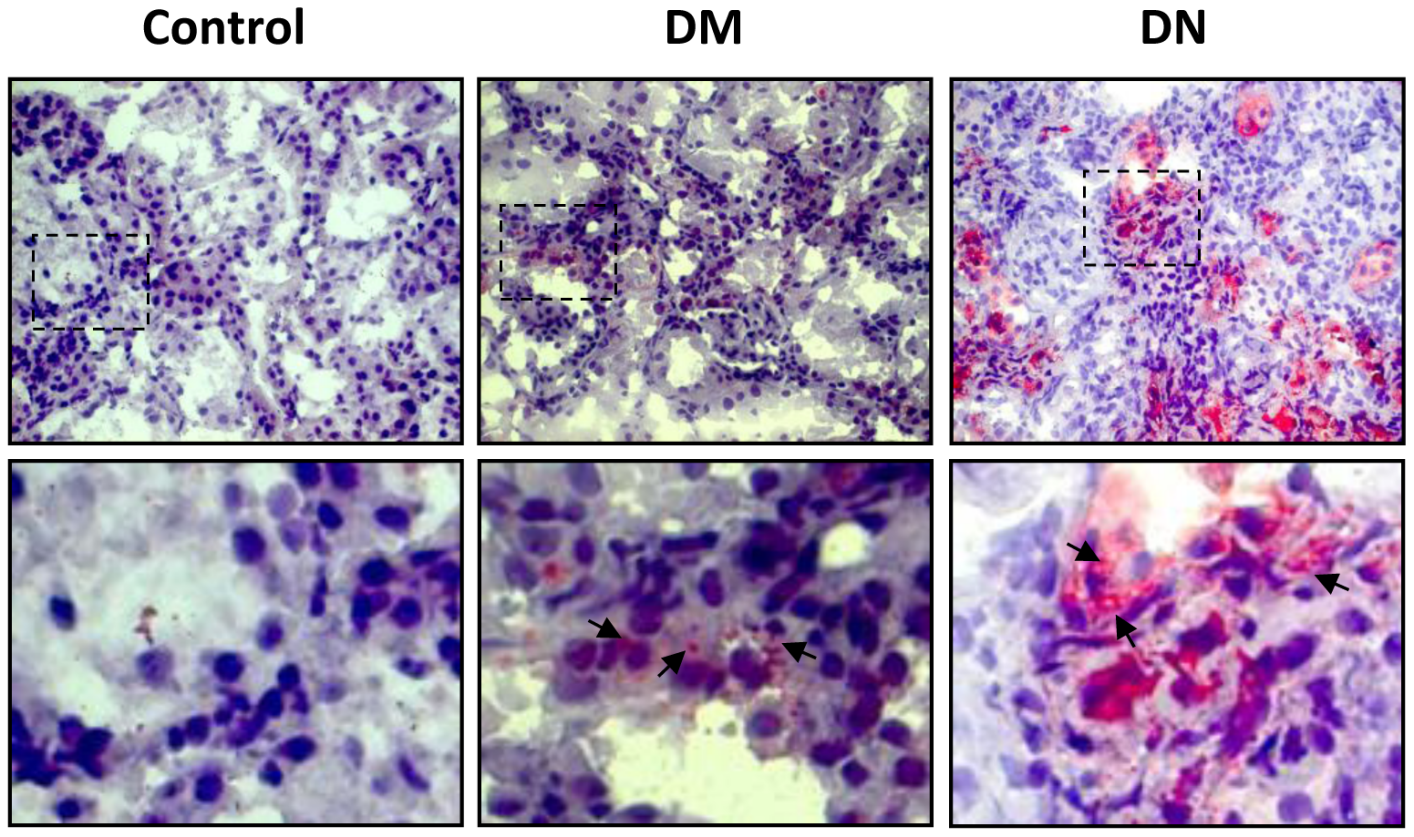
Oil Red O staining of cholesterol in renal tissues. Oil Red O staining of cholesterol in renal tissues from control, diabetic (DM), and diabetic nephropathy (DN) mice. Original magnification x400. Box area is enlarged to compare cholesterol accumulation (denoted by arrows) between control and diabetic mice (lower panels).

**Figure 5:**
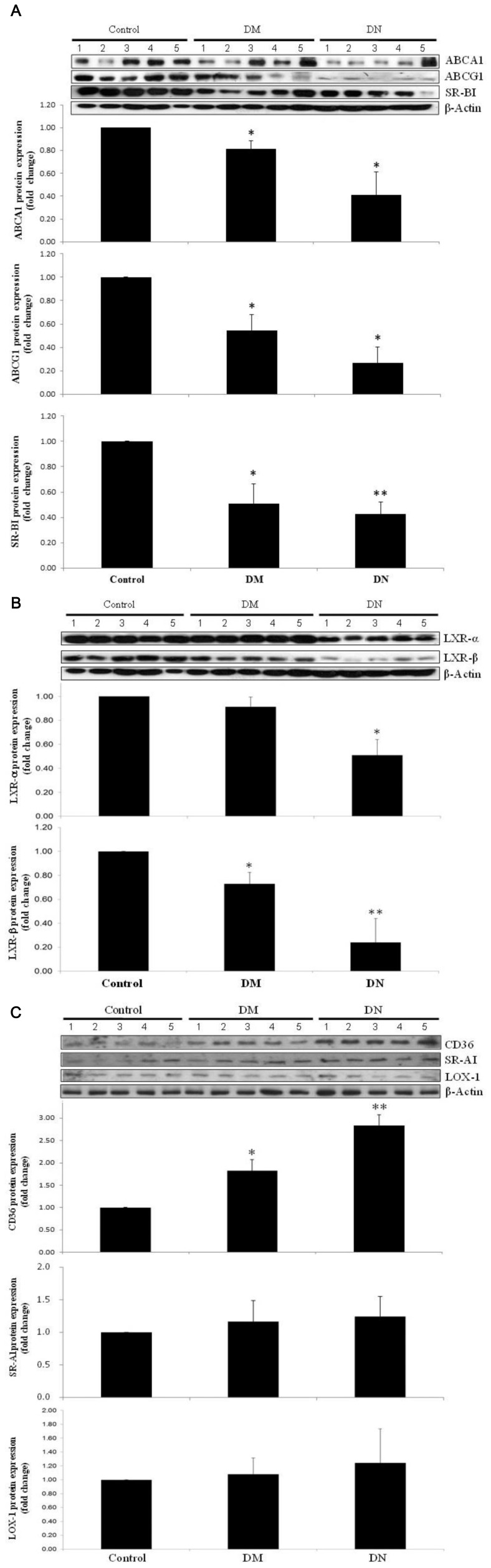
Renal expression of cholesterol uptake and efflux proteins. Protein expression of ABCA1, ABCG1 and SR-BI (A), LXR-α, LXR-β (B) and CD36, SR-AI, and LOX-1 (C) in kidneys from control, diabetic (DM), and diabetic nephropathy (DN) mice. Relative target protein band intensities were normalized against its *β*-actin and were presented as means + SD by densitometric analysis from 5 mices per group. *p<0.05 and **p<0.01 vs. Control.

*In vivo* studies were performed to investigate the renal expression of cholesterol transporters in diabetic mice. Plasma lipids were only significantly increased in the diabetic mice with nephropathy (DN) (Table 1). Glomerular abnormalities and tubulo-interstitial changes were noted in mice with diabetic nephropathy. Histological examination revealed that ABCA1, ABCG1 and SR-BI were mainly expressed in renal tubules, and there was a marked reduction in all three cholesterol transporters in diabetic mice especially in the group with nephropathy (Figure 3). Oil Red O staining revealed that lipid droplets accumulated particularly at the renal tubules and was most marked in the diabetic nephropathy group (Figure 4). Protein expressions of ABCA1, ABCG1 and SR-BI were significantly reduced in the kidneys from diabetic animals with the greatest reduction seen in mice with nephropathy (Figure 5A). Interestingly, the expression of LXR-α and LXR-β, the nuclear receptors that act as important positive regulators of ABCA1 and ABCG1, were also significantly reduced in the kidneys of diabetic mice (Figure 5B). On the other hand, evaluation of the scavenger receptors that mediated the uptake of lipoproteins including CD36, SR-AI, and lectin-like oxidized lipoprotein receptor-1 (LOX-1) showed that only the expression of CD36 was increased in diabetic animals (Figure 5C).

**Table 1:** Plasma glucose and lipids in control and diabetic mice.

	Control	DM	DN
Weight (g)	26.6±2.1	33.9±3.0**	26.1±1.4
Glucose (mmol/L)	9.1±0.6	30.4±0.6**	32.2±2.0**
Triglyceride (mmol/L)	0.60±0.27	0.50±0.22	1.36±0.60*
Cholesterol (mmol/L)	2.89±0.34	3.08±0.41	3.92±0.74*
Urine albumin to creatinine ratio	3.02±0.61	4.28±1.28	16.81±4.69*

Data are the means ± SD. *p<0.05, **p<0.01 vs control mice.

## Discussion

Efflux of free cholesterol from cells is an early step of reverse cholesterol transport. The role of the cholesterol transporters ABCA1, ABCG1 and SR-BI in macrophage cholesterol efflux has been extensively studied, and it has been shown that impaired cellular cholesterol efflux is associated with atherosclerosis in humans [12]. The role of cholesterol transporters and cellular cholesterol efflux in renal disease is less clear. Previous studies have shown that mesangial cell express ABCA1 and tubular cells express both ABCA1 and SR-BI [13], [16]. Although SR-BI can mediate bi-directional cholesterol flux for instance, in the liver, Zager et al. have demonstrated that SR-BI predominantly functions as a cholesterol efflux pathway in tubular cells [16]. We have shown for the first time that all three cholesterol transporters are expressed in human mesangial cells and proximal tubular epithelial cells, and the expression of these cholesterol transporters can be suppressed under high glucose conditions *in vitro*. Song et al. recently reported that without the factor of high fat, high glucose reduced ABCG1 expression in mouse mesangial cells [22]. However, they did not find any changes in ABCA1 expression, and this might be due to differences in cell lines and experimental conditions used.

Studies in nephrotic rats have shown that increased kidney tissue cholesterol was associated with reduced renal expression of SR-BI whereas the expression of ABCA1 was either unchanged or decreased [23], [24]. In contrast, accumulation of lipid in the remnant kidney in rats with chronic renal failure induced by 5/6 nephrectomy was accompanied by up-regulation of both ABCA1 and SR-BI [25]. Only the role of ABCA1 has previously been examined in mouse models of type 1 diabetes and renal expression of ABCA1 was reduced [17], [26]. Proctor et al. suggested that the accumulation of renal lipids was due to increased cholesterol and fatty acid synthesis as well as reduced cholesterol efflux mediated by ABCA1 [26]. However, it is not clear whether the reduction in ABCA1 expression might be compensated by the up-regulation of other cholesterol transporters.

Our study has provided new data on ABCG1 and SR-BI in diabetic nephropathy. Firstly, we have shown that in addition to ABCA1, ABCG1 and SR-BI were also expressed in both mesangial cells and tubular cells, and we have evaluated the distribution of these cholesterol transporters in the kidneys. All three cholesterol transporters were most highly expressed in proximal tubules of mouse kidneys, and were weakly detected in the glomeruli. Secondly, inducing diabetes with streptozotocin significantly reduced renal expression of not only ABCA1 but also ABCG1 and SR-BI, and these changes preceded the development of diabetic nephropathy. Since all three transporters were involved, cellular cholesterol efflux to both apo AI and HDL would be affected. Defects in cholesterol export pathway in renal cells could therefore promote cholesterol accumulation. In our study, diabetic mice with nephropathy had the lowest level of expression of all three cholesterol transporters and this was accompanied by an increase in CD36. Hence, an increase in modified lipoproteins uptake by CD36 coupled with reduction in lipid efflux pathways would contribute to the increased amount of lipid droplets and foam cells in glomeruli and tubulointerstitium of diabetic mice.

In conclusion, the expression of ABCA1, ABCG1 and SR-BI in mesangial cells and tubular cells were significantly decreased under hyperglycemic conditions *in vitro*, and renal expression of these cholesterol transporters was reduced in diabetic mice with nephropathy. Our results suggest that impaired cellular cholesterol efflux in the kidney might contribute to the development of diabetic nephropathy.

## Supporting Information

Figure S1 **Basal expression of cholesterol transporters in mesangial cells and tubular HK-2 cells.** Relative target protein band intensities of ABCA1 (a), ABCG1 (b) and SR-BI (c) were normalized against its β-actin respectively and were presented as means + SD by densitometric analysis from 3 separate experiments.
(DOCX)

Figure S2 **Cellular cholesterol efflux of mesangial cells and tubular HK-2 cells.** Cellular cholesterol efflux from mesangial cells and tubular cells to HDL, HDL_2_ and HDL_3_ was presented as means + SD from 3 separate experiments in duplicate. No significant difference was seen between cholesterol efflux to HDL, HDL_2_ and HDL_3_.
(DOCX)

Figure S3 **Cellular cholesterol efflux of mesangial cells and tubular HK-2 cells under different concentrations of glucose.** Cellular cholesterol efflux to apo AI and HDL from mesangial cells (a) and tubular cells (b) cultured under different conditions of glucose concentrations were presented as means + SD from 3 separate experiments in duplicate, *p<0.05 and **p<0.01 vs. cells with 5 mM glucose in culture medium.
(DOCX)
