# Supplementary material for: Cellular Cholesterol Transport Proteins in Diabetic Nephropathy
Source: PLoS One. 2014 Sep 2;9(9):e105787. doi: 10.1371/journal.pone.0105787 (PMC4152117; doi:10.1371/journal.pone.0105787)
Supplement: Figure S1 — Basal expression of cholesterol transporters in mesangial cells and tubular HK-2 cells. Relative target protein band intensities of ABCA1 (a), ABCG1 (b) and SR-BI (c) were normalized against its β-actin respectively and were presented as means + SD by densitometric analysis from 3 separate experiments. (DOCX) [file pone.0105787.s001.docx]

**Supporting information**

**Figure S1. Basal expression of cholesterol transporters in mesangial cells and tubular HK-2 cells.** Relative target protein band intensities of ABCA1(a), ABCG1(b) and SR-BI(c) were normalized against its β-actin respectively and were presented as means + SD by densitometric analysis from 3 separate experiments.


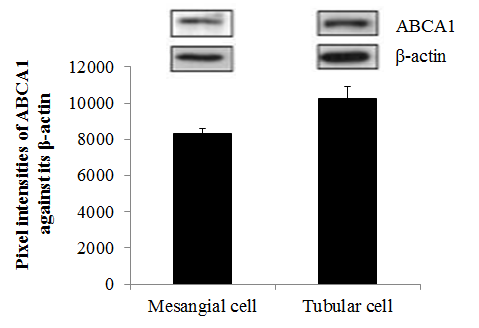
S1a


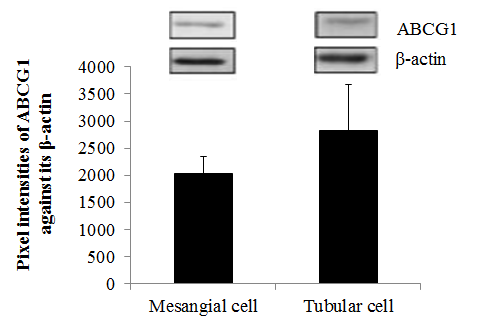
S1b


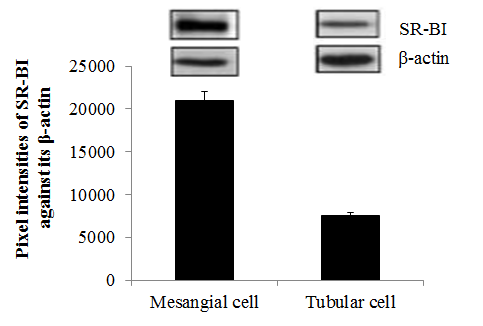


S1c
