# Supplementary material for: Cellular Cholesterol Transport Proteins in Diabetic Nephropathy
Source: PLoS One. 2014 Sep 2;9(9):e105787. doi: 10.1371/journal.pone.0105787 (PMC4152117; doi:10.1371/journal.pone.0105787)
Supplement: Figure S2 — Cellular cholesterol efflux of mesangial cells and tubular HK-2 cells. Cellular cholesterol efflux from mesangial cells and tubular cells to HDL, HDL2 and HDL3 was presented as means + SD from 3 separate experiments in duplicate. No significant difference was seen between cholesterol efflux to HDL, HDL2 and HDL3. (DOCX) [file pone.0105787.s002.docx]

**Figure S2. Cellular cholesterol efflux of mesangial cells and tubular HK-2 cells.** Cellular cholesterol efflux from mesangial cells and tubular cells to HDL, HDL_2_ and HDL_3_ was presented as means + SD from 3 separate experiments in duplicate. No significant difference was seen between cholesterol efflux to HDL, HDL_2_ and HDL_3_.

**
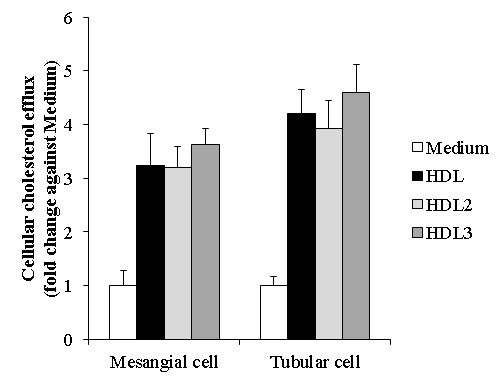
**
