# Supplementary material for: Cellular Cholesterol Transport Proteins in Diabetic Nephropathy
Source: PLoS One. 2014 Sep 2;9(9):e105787. doi: 10.1371/journal.pone.0105787 (PMC4152117; doi:10.1371/journal.pone.0105787)

**Figure S3. Cellular cholesterol efflux of mesangial cells and tubular HK-2 cells under different concentrations of glucose.** Cellular cholesterol efflux to apo AI and HDL from mesangial cells (a) and tubular cells (b) cultured under different conditions of glucose concentrations were presented as means + SD from 3 separate experiments in duplicate, **p*<0.05 and ***p*<0.01 vs. cells with 5mM glucose in culture medium.

S3a


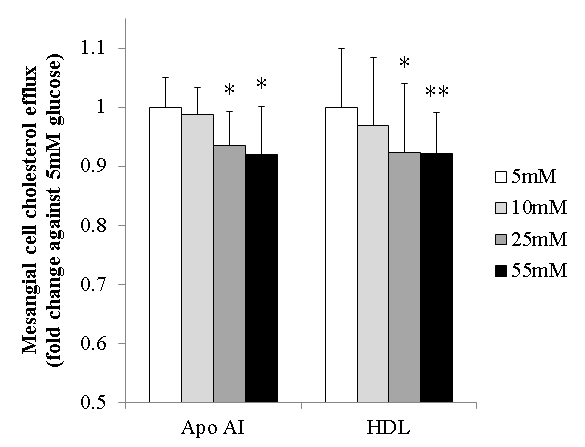


S3b


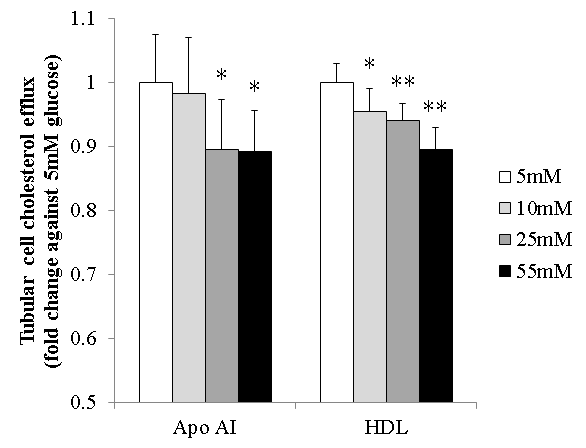

Supplement: Figure S3 — Cellular cholesterol efflux of mesangial cells and tubular HK-2 cells under different concentrations of glucose. Cellular cholesterol efflux to apo AI and HDL from mesangial cells (a) and tubular cells (b) cultured under different conditions of glucose concentrations were presented as means + SD from 3 separate experiments in duplicate, *p<0.05 and **p<0.01 vs. cells with 5 mM glucose in culture medium. (DOCX) [file pone.0105787.s003.docx]
